# Collaborative Research on Ageing in Europe project’s aims and main results

**DOI:** 10.1186/1753-6561-7-S4-S3

**Published:** 2013-08-23

**Authors:** Matilde Leonardi, Alberto Raggi, Rui Quintas

**Affiliations:** 1 Neurology, Public Health and Disability Departmental Unit, Foundation IRCCS, Institute of Neurology Carlo Besta, Milan, Italy

## 

The FP7 Collaborative Research on Ageing in Europe project (COURAGE) (http://www.courageproject.eu) collected data on the determinants of health and disability in an ageing population, with specific tools for the evaluation of the role of built environment and social networks on health, disability, quality of life and well-being. The aims of the project were to develop a survey protocol for European studies on ageing and determinants of disability in ageing, demonstrating its reliability and validity, and to demonstrate substantial innovations in ageing survey methodology, providing a cross-population analysis and a baseline for European and international longitudinal data collection.

The project’s fieldwork was conducted, from May 2011 to March 2012, on a sample of 10,800 persons from Finland, Poland and Spain. Mean age was 59.27 for Finland, 57.62 for Poland and 60.44 years for Spain.

On the whole sample a trend of increase in functioning difficulties with age and with levels of household wealth was observed, with older subjects and those with lower wealth reporting more difficulties. Quality of life is perceived as better in Finland and in Spain than in Poland. The levels of quality of life decrease with the increase of age, and in Poland this decreasing is significantly higher.

People from Finland showed the highest well-being, and those from Poland the lowest. Life evaluation worsened along the life span, whereas the affect tended to improve: positive affect increased and negative affect decreased in Finland and Spain. In Poland negative affect increased with age.

Worse social networks are associated with higher age, while higher level of education is associated with better social networks. Furthermore, better levels of social networks are related with living in rural places, for the three countries. Reported good or very good health is associated with a better persons-environment interaction; people younger than 50 perceived their neighbourhood environment as more usable. Country-specific differences were found: in general, Polish respondents reported worse person-environment interactions, and Spanish better interactions. In the objective evaluation of built environment was found that environment was assessed as more facilitative in Spain than in Finland.

In conclusion, the newly developed and validated COURAGE Protocol for Ageing Studies has proven to be a valid tool for collecting comparable data in ageing populations. The COURAGE Project created valid and reliable scientific evidence for disability and ageing research and policy development that showed cross-country comparability. It is therefore recommended that future studies exploring determinants of health and disability in ageing use COURAGE-derived methodology.

